# Health-related quality of life and physical recovery after a critical illness: a multi-centre randomised controlled trial of a home-based physical rehabilitation program

**DOI:** 10.1186/cc10265

**Published:** 2011-06-09

**Authors:** Doug Elliott, Sharon McKinley, Jennifer Alison, Leanne M Aitken, Madeleine King, Gavin D Leslie, Patricia Kenny, Penny Taylor, Rachel Foley, Elizabeth Burmeister

**Affiliations:** 1 Faculty of Nursing, Midwifery and Health, University of Technology, Sydney, 15 Broadway, Ultimo, 2007, Australia; 2 University of Technology, Sydney and Northern Sydney Local Health Network, Sydney,15 Broadway, Ultimo, 2007, Australia; 3 Faculty of Health Sciences, The University of Sydney, 75 East Street, Lidcombe, 2141, Australia; 4 Princess Alexandra Hospital and Griffith University, 199 Ipswich Road, Woolloongabba, 4102, Australia; 5 School of Psychology, University of Sydney, Fisher Road, Sydney, 2006, Australia; 6 School of Nursing and Midwifery, Curtin Health Innovation Research Institute, Curtin University and Royal Perth Hospital, Kent Street, Bentley, 6102, Australia; 7 Centre for Health Economics Research and Evaluation, University of Technology, Sydney, 15 Broadway, Ultimo, 2007, Australia; 8 Critical Care Nursing Professorial Unit, University of Technology, Sydney and Northern Sydney Local Health Network, 15 Broadway, Ultimo, 2007, Australia; 9 Nursing Practice and Development Unit, Princess Alexandra Hospital, 199 Ipswich Road, Woolloongabba, 4102, Australia

## Abstract

**Introduction:**

Significant physical sequelae exist for some survivors of a critical illness. There are, however, few studies that have examined specific interventions to improve their recovery, and none have tested a home-based physical rehabilitation program incorporating trainer visits to participants' homes. This study was designed to test the effect of an individualised eight-week home-based physical rehabilitation program on recovery.

**Methods:**

A multi-centre randomised controlled trial design was used. Adult intensive care patients (length of stay of at least 48 hours and mechanically ventilated for 24 hours or more) were recruited from 12 Australian hospitals between 2005 and 2008. Graded, individualised endurance and strength training intervention was prescribed over eight weeks, with three physical trainer home visits, four follow-up phone calls, and supported by a printed exercise manual. The main outcome measures were blinded assessments of physical function; SF-36 physical function (PF) scale and six-minute walk test (6MWT), and health-related quality of life (SF-36) conducted at 1, 8 and 26 weeks after hospital discharge.

**Results:**

Of the 195 participants randomised, 183, 173 and 161 completed the 1, 8 and 26 weeks assessments, respectively. Study groups were similar at Week 1 post-hospital; for the intervention and control groups respectively, mean norm-based PF scores were 27 and 29 and the 6MWT distance was 291 and 324 metres. Both groups experienced significant and clinically important improvements in PF scores and 6MWT distance at 8 weeks, which persisted at 26 weeks. Mixed model analysis showed no significant group effects (*P *= 0.84) or group by time interactions (*P *= 0.68) for PF. Similar results were found for 6MWT and the SF-36 summary scores.

**Conclusions:**

This individualised eight-week home-based physical rehabilitation program did not increase the underlying rate of recovery in this sample, with both groups of critically ill survivors improving their physical function over the 26 weeks of follow-up. Further research should explore improving effectiveness of the intervention by increasing exercise intensity and frequency, and identifying individuals who would benefit most from this intervention.

**Trial registration:**

Australia and New Zealand Clinical Trials Register ACTRN12605000166673

## Introduction

A critical illness requiring admission to a general intensive care unit (ICU) affects approximately 119,000 adult Australians per year [1]. While survival rates approximate 89% at hospital discharge [2], functional recovery for individuals is delayed often beyond six months post-discharge [3,4]. Physical de-conditioning and neuromuscular dysfunction [5,6] as well as psychological sequelae [7] are common, adding to the burden of illness for survivors, carers, the health care system and broader society [8].

Reviews of numerous observational studies confirm delayed recovery in health-related quality of life (HRQOL), [for example, 3, 4, 9] and anxiety (12-43%) [10], depression (median 28%) [11] and distress (including post-traumatic stress symptoms; 5-64%) [12] are prevalent. While significant sequelae therefore exist for a substantial proportion of critical illness survivors, little evidence is currently available to support specific interventions for improving their recovery [8,13], with very few published interventional studies focusing on the post-hospital discharge period; for example, follow-up clinics [14], a patient self-managed rehabilitation manual [15]. No studies have tested the effects of home-based rehabilitation involving trainer visits on patient recovery.

We proposed that a focused home-based approach to physical rehabilitation in addition to usual community-based health services, would improve the HRQOL and recovery of individuals surviving a critical illness. The rehabilitation program for this cohort reflected similar successful programs in cardiac and respiratory disease [16,17] by optimising functional recovery, particularly during the first few months after a critical illness.

## Materials and methods

### Design, hypothesis and secondary aims

A multi-centre randomised controlled trial (RCT) design was used to test the effects of an eight-week home-based rehabilitation program on HRQOL and physical function for individuals who survived a critical illness. The primary research hypothesis was: Survivors of a critical illness who participated in the physical rehabilitation program have better physical function, as measured by a difference of 10 points on the Physical Functioning (PF) scale of the Short-Form-36 Health Survey (SF-36), when compared to those who received usual care at eight weeks after hospital discharge (short-term effect); and that this group difference would persist at 26 weeks (long-term effect). Secondary aims were to test the program effectiveness for: improvement in other domains of HRQOL (Component Summary scores of SF-36 [18]); and better functional exercise capacity, measured by the six-minute walk test (6MWT) [19].

The study protocol was published previously [20] and the flow of participants reflects the CONSORT statement [21]. Human Research Ethics Committee approvals were obtained from each of the recruitment site hospitals and the universities of the investigators. A safety protocol ensured assessor and trainer safety during home visits [20].

### Participants and sample size

Participants were initially recruited from ICUs in Sydney and Brisbane (from four teaching and three district hospitals) with other recruitment sites added progressively from Sydney and Perth (two teaching, two district and one private hospital). Sample size was calculated for the SF-36 PF scale for a two-sided hypothesis test with a Type I error rate of 0.05 and a Type II error rate of 0.20 (80% power). The clinically important difference and the standard deviation estimates used were based on our pilot data [22,23] and reports for similar cohorts and contexts [15,24-26].

At baseline (one week post-hospital discharge), we anticipated that both groups would have mean PF scores of 45. We postulated that the control group would improve by 5 points at eight weeks, with the intervention group improving by 15 points, giving a difference of 10 points between the two study groups using the traditional non-normalised raw score for SF-36. Using the 10 items that comprise the PF scale of SF-36, this improvement represents a change from 'limited a lot' to 'limited a little' on three items in the scale, for example in climbing stairs or walking particular distances. These changes reflect significant clinical improvement in physical function [27].

A sample of 100 patients per study group was required to detect this difference, assuming similar group variance (SD = 25) [22,28]. We planned to over-enrol by 20% to account for losses to follow-up (10% study attrition [15]; 10% mortality at six-months post-hospital discharge following a critical illness) [1,29]. The total planned recruitment was therefore 240.

To be eligible for enrolment, participants: 1) were aged 18 years or older; 2) had an ICU length of stay (LOS) of ≥48 hours; 3) received mechanical ventilation for ≥24 hours; 4) were discharged home to self-care or carer (non-institutional care); 5) resided within the hospitals' local geographical areas to enable home visits (an approximately 50 km radius); 6) had no neurological, spinal or skeletal dysfunction preventing participation in physical rehabilitation; 7) were not receiving palliative care; 8) had no organised rehabilitation related to ongoing chronic disease management (for example, pulmonary rehabilitation, cardiac rehabilitation); and 9) were cognitively able to complete the self-report measures and comply with physical testing instructions.

### Randomisation

Eligible patients were approached following ICU discharge; informed voluntary consent was either obtained at that time or following agreement to be contacted at home after hospital discharge. After participant consent, the site project officer contacted an independent telephone randomisation service for the participant study number and group allocation. The service used a blocked random allocation sequences (one for each recruitment site) generated using SAS software [30] by our study statistician (MK).

### Intervention

Participants in the control group received usual community-based care after hospital discharge (for example, visits to their general practitioner), as well as the three study assessment visits, but no other placebo or sham interventions. Following the first home-based assessment, participants randomised to the intervention group received an eight-week home-based physical rehabilitation program that focused on strength training and walking. A qualified trainer (physiotherapist, exercise physiologist or registered nurse with additional specific training for this project) visited participants at home in weeks 1, 3 and 6 to provide individualised verbal and written instructions on their planned exercise program. Each home visit session was 60 to 90 minutes in duration. The trainer also telephoned intervention group participants in weeks 2, 4, 5 and 7 to monitor their progress.

The program reflected standard approaches for improving muscle strength and endurance within cardiac and pulmonary rehabilitation settings [31,32]. Exercise prescription and supervised physical rehabilitation training involved graded, individualised endurance and strength training designed by a pulmonary rehabilitation physiotherapist. Training focused initially on walking (endurance training) and lower limb exercises (strength training). As participants progressed, core stabilisation and upper limb exercises were introduced. The remaining two trainer home visits and telephone contacts in non-visit weeks assessed participant progress and compliance, prescribed progression and reinforced the exercise program [20].

An illustrated exercise manual supported the participant's training and graded progression, structured in three parts: Part 1 described how to gauge exercise intensity based on a level of 'moderate' to 'somewhat heavy' perceived exertion (score of 3 to 4 on the modified Borg Scale) [33] and also provided information about participant safety; Part 2 provided a detailed exercise program; and Part 3 described how to progress the endurance and strength training. The exercise program consisted of five components-endurance exercise (walking), lower and upper limb strengthening, core stabilisation, flexibility, and stretches. A total of 16 different exercises were numbered, named, illustrated and described, to facilitate participant-trainer communication and exercise progression. This included four stretching, three flexion, and three core stabilisation exercises, which were included in the trainer's exercise prescription based on assessment of the participant's capabilities and needs.

### Endurance (walk) training

Exercise prescription for endurance training was based on the results of each participant's 6MWT during the Week 1 assessment visit. Training intensity commenced at 80% of peak walking speed. Extra activities were prescribed based on a level of perceived exertion of 3 to 4 using the modified Borg scale [33]. A walk-rest-walk approach was used, with the duration of walking varying according to the participant's ability and condition; 12 levels of walking were described ranging from 1 to 60 minutes. Participants worked towards an optimal goal of training for five days per week for 20 to 30 minutes of walking by the end of the program.

### Strength training

This training included upper (biceps, triceps, shoulder abductors/adductors) and lower limb (quadriceps, hamstrings, hip abductors and extensors) muscle groups. Initial prescription was one set of eight-repetition maximum (8RM) for each activity, progressing to three sets. Further progression was based on increasing weight (0.25 to 1.5 kg for arm exercises using cans of food or bags of rice), and increasing the step height or weight for lower limb exercises. Levels of progression were described in the exercise manual.

### Outcomes

Each participant was assessed in-home within one week of hospital discharge by an assessor blind to group allocation, with follow-up assessments at 8 and 26 weeks post-discharge. The primary outcome measure was the physical functioning of study participants, using the SF-36 PF scale (version 2) [18]. SF-36 has demonstrated reliability, validity and responsiveness in the post-ICU population [34], and is the most common instrument used for assessing health status in this patient cohort [3,4,8,9].

Secondary outcome measures were exercise capacity measured using the 6MWT [19]; and HRQOL. The 6MWT was performed twice at each assessment, to account for any learning effect, with the best result recorded for analysis. During the 6MWT, participants were directly observed and monitored continuously by the assessor using a portable pulse oximeter (measuring pulse rate and oxygen saturation), with their exertion level assessed and documented during the test [19] using the Borg perceived exertion scale [33]. Additional aspects of HRQOL were measured using the Physical Component Summary (PCS) and Mental Component Summary (MCS) scales, which combine information from all eight domains of SF-36 [18].

### Statistical methods

Data were entered into a purpose-built MS Access database at the three coordinating sites; monthly site reports on enrolment, randomisation and participant follow-up were submitted to one central site and monthly summaries of the whole cohort reviewed by our team. Analysis was by intention-to-treat, and was conducted for the primary outcome (SF-36 physical functioning; PF) and three secondary outcomes, the 6MWT distance, the physical component summary (PCS) and the mental component summary (MCS) scores of SF-36. The SF-36 scales (PF, PCS and MCS) were calculated as per the user's manual [18]. The eight raw domain scores were transformed to a score range of 0 to 100 (a higher score represents better functioning/HRQOL). Domain scores were then further summarised into PCS and MCS scores using z-scores with each domain mean and standard deviation derived from Australian normative data [35,36]. The component aggregate score was calculated by summing the weighted z-scores using factor score coefficients from normative population data as weight. The aggregate component scores and the PF domain z-score were then converted to norm-based scores (NBS) as: NBS = 50 + (z-score × 10) [36]. Norm-based scores are interpreted in relation to the population mean of 50 and a standard deviation of 10. Baseline characteristics of both groups were described in terms of percentages for categorical variables and mean and standard deviation for continuous variables.

Study hypotheses were tested with mixed linear regression models estimated by residual maximum likelihood using SAS Proc Mixed [30]. Fixed effects were estimated for randomisation group (to test for differences between groups at eight weeks), time (to test for change from 8 to 26 weeks), and a group by time interaction (to test for differences in change by group), with the baseline level of the outcome variable included as a covariate. Each model included a random person specific intercept to account for within person correlation. Patient characteristics considered to be potential confounders (age, gender, APACHE II scores, days in ICU, days in hospital) and site were included as covariates. Covariates were retained if: 1) there was evidence of confounding (estimates of treatment effect differed markedly between adjusted versus unadjusted models); 2) they explained significant variation in the outcome; 3) they improved the precision of the estimates of treatment effect. The F-test was used to test for the significance of effects and the adjusted mean levels of each outcome variable were calculated for each group at 8 and 26 weeks (adjusted for baseline levels). As a secondary descriptive analysis, individual change scores were calculated between baseline (Week 1 post-hospital discharge) and at 8 and 26 weeks. Within-group effect size was calculated as mean change from baseline/standard deviation at baseline, and between-group effect size was calculated as the difference between groups in mean change from baseline divided by the pooled standard deviation for change [37].

## Results

**Figure 1:**
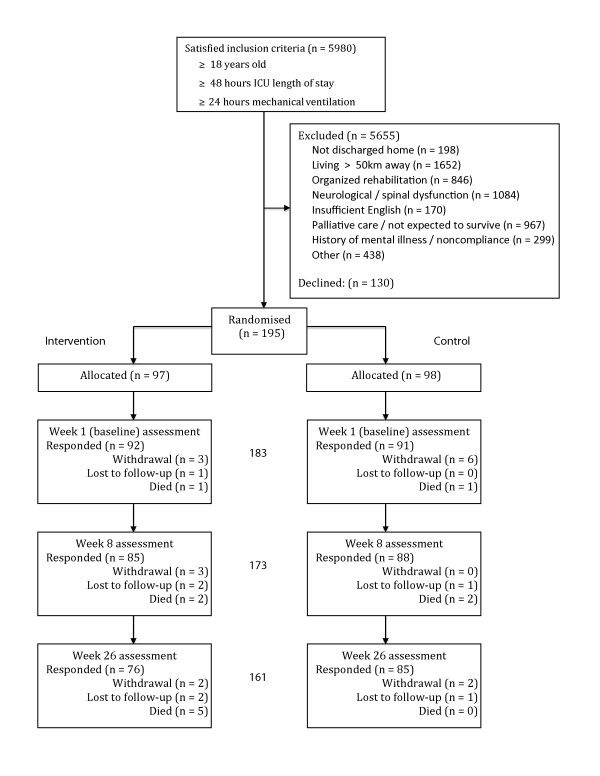
**Consort flow diagram of patient recruitment and retention**.

Study recruitment occurred from June 2005 to August 2008, with final follow-up data collection completed in February 2009. Of the 5,980 patients screened, 5,655 were excluded; the main reasons were excessive distance from the study sites precluding home visits, neurological or spinal dysfunction precluding physical training, and palliative care/not expected to survive ICU admission (Figure 1). Of the 195 patients randomised during their post-ICU hospitalisation, 93% provided primary outcome data at week 1 post-hospital discharge. Their subsequent response rate was 95% (97% control and 92% intervention) at 8 weeks and 88% (93% of control and 85% of intervention) at 26 weeks. Eleven patients died and 16 withdrew during the study period (Figure 1).

Characteristics for the intervention and control groups were similar at baseline (Table 1). The mean age of the sample was 57 years, and 61% were male. Both groups had a median ICU length of stay of 6 days and median hospital length of stay of 18.5 days. The overall median period of mechanical ventilation was 90 hours, with a mean Day 1 APACHE II score of 19.5. Fifty-five percent of participants had a non-operative diagnosis. The most prevalent APACHE III diagnostic groups on admission were gastrointestinal (30%), respiratory (24%) and cardiovascular (20%), with 8% sepsis and 6% trauma diagnoses. The baseline (Week 1) mean norm-based PF scores were 27.1 and 28.8 for the intervention and control groups respectively, and the 6MWT distance was 291 and 324 metres (Table 1). Physical functioning at baseline did not differ significantly between those with complete and incomplete data (*P *= 0.86).

**Table 1:** Sample baseline characteristics

Variable		Control (*n *= 91)	Intervention (*n *= 92)
Age	mean (sd)	57.5 (15.1)	57.2 (17.0)
Gender	%	M: 61	M: 62
APACHE II	mean (sd)	19.5 (7.2)	19.4 (12.6)
MV hours	mean (sd)	135 (117)	142 (159)
	median^c ^(IQR)	91 (48-179)	76 (41-180)
ICU LOS days	mean (sd)	8.6 (7.5)	9.4 (8.7)
	median (IQR)	6 (4-10.5)	6 (4-11)
Hospital LOS days	mean (sd)	23.2 (16.9)	24.8 (20.4)
	median (IQR)	18.5 (12-27.5)	18.5 (12-30)
SF-36 PF^a^	mean (sd)	28.8 (10.2)	27.1 (12.3)
6MWT distance metres^a^	mean (sd)	324 (143)	291 (129)
SF-36 PCS^b^	mean (sd)	32.7 (8.6)	31.7 (10.0)
SF-36 MCS^b^	mean (sd)	39.8 (13.5)	36.7 (15.1)

APACHE, acute physiology and chronic health evaluation; LOS, length of stay; MCS, mental components summary; MV, mechanical ventilation hours; PCS, physical components summary

^a^Measured at one week post-hospital discharge

^b^Norm-based score; Australian normative population mean = 50; standard deviation = 10

^c^Medians included only for those variables with skewed distributions

There were no significant group effects or group by time interactions (see Table 2) for PF, and no significant covariates after adjusting for baseline PF. This was also the case for 6MWT, MCS and PCS (Table 2). The time effect was significant for PF (*P *= 0.034) and 6MWT (*P *= 0.0003), but not for PCS (*P *= 0.06) or MCS (*P *= 0.97). Both control and intervention groups showed similar improvements between Week 1 and Week 8, and Week 1 and Week 26 for the PF and 6MWT, and the PCS and MCS (see Table 3). Clinically important change scores of 12 (control) and 13 (intervention) for the mean PF were noted at eight weeks. The change scores between weeks 1 to 26 were 14 and 15 respectively, with little additional improvement from the eight-week assessment. Other domains for SF-36 were also comparable between groups at all time points (Table 4 details the domain scores for the two groups). Change scores for 6MWT distance were 80 and 89 metres at 8 weeks, and 116 and 126 metres at 26 weeks, for the control and intervention groups respectively (Table 3). Effect sizes for the impact of the intervention were very small for all measures at 8 and 26 weeks (Table 3), consistent with the mixed linear regression models (Table 2).

**Table 2:** Mixed linear regression model: mean outcomes adjusted for Week 1 (baseline) levels

Outcome	Week	Control	Treatment	*P*-value
**Physical functioning**	8	41.0	39.9	Group0.84
	26	41.8	42.6	Time0.034
				Group × time 0.68
**Six Minute Walk Test distance**	8	395.6	402.5	Group0.92
	26	431.4	428.3	Time0.0003
				Group × time 0.55
**Physical component summary**	8	42.5	40.7	Group0.39
	26	43.2	42.7	Time0.06
				Group × time 0.37
**Mental component summary**	8	47.1	46.9	Group0.95
	26	47.0	47.0	Time0.97
				Group × time 0.89

**Table 3:** Mean change from baseline and effect size at 8 and 26 weeks following discharge

	Control (95% CI)	Effect Size^a^	Intervention (95% CI)	Effect Size^a^	Difference (95% CI)	Effect Size^b^
**8 weeks**						
**PF^d^**	12.2 (9.9,14.5)	1.17	12.9 (10.7,15.1)	1.02	0.7 (-2.5,3.8)	**0.07**
**6MWT**	80.3 (52.3,108.3)	0.55	88.7 (62.6,114.8)	0.69	8.4 (-29.6, 46.4)	**0.07**
**PCS^c^**	9.9 (7.6,12.2)	1.10	8.6 (6.6,10.5)	0.87	-1.3 (-4.3, 1.7)	**-0.14**
**MCS^c^**	7.8 (4.8,10.9)	0.59	9.7 (6.4,12.9)	0.66	1.8 (-2.6, 6.2)	**0.13**
**26 weeks**						
**PF**	13.7 (11.4,16.0)	1.32	14.6 (11.7,17.6)	1.16	0.9 (-2.7, 4.6)	**0.08**
**6MWT**	116.2 (85.6,146.8)	0.80	125.8 (98.7,152.9)	0.98	9.6 (-31.4, 50.5)	**0.08**
**PCS^c^**	10.6 (8.4,12.8)	1.18	10.9 (8.2,13.6)	1.11	0.3 (-3.2, 3.7)	**0.03**

MCS^c^	8.1 (5.0,11.2)	0.61	9.6 (6.1,13.1)	0.65	1.5 (-3.1, 6.2)	0.10

6MWT, six minute walk test distance (metres); CI, confidence interval; MCS, mental component summary; PCS, physical component summary; PF, physical functioning

^a^Effect size = mean change from baseline/standard deviation at baseline

^b^Effect size = (Intervention mean change-Control mean change)/ pooled standard deviation for change

^c^Norm-based score for Australian normative population mean = 50, standard deviation = 10.

**Table 4:** Mean norm-based^a^SF-36 scores by assessment time point and group

SF-36 Domains	Week 1		Week 8		Week 26	
**Groups**	**C**	**I**	**C**	**I**	**C**	**I**

Physical function	29.1	27.3	41.0	39.9	41.8	42.6
Role function-physical	25.5	25.1	38.0	38.2	40.9	42.1
Bodily pain	43.0	38.7	49.0	46.7	46.9	44.5
General health	43.5	41.7	46.0	44.7	45.3	44.8
Vitality	38.1	36.0	46.9	45.5	47.0	47.6
Social function	30.1	27.9	44.9	43.0	44.5	44.5
Role function-emotional	32.0	28.0	42.4	41.1	42.9	43.6
Mental health	43.4	40.1	48.2	48.0	48.1	48.4
Physical component Summary	33.0	31.6	42.7	40.5	42.9	42.8
Mental component summary	40.0	36.6	47.5	46.9	47.2	48.0

C, control; I, intervention.

^a^Norm-based scores for domain and summary scores are calculated from raw scores using population-based data for the Australian population; scores are interpreted in relation to a population mean of 50 and a standard deviation of 10.

## Discussion

### Major findings

Our main findings were that this home-based rehabilitation intervention had no significant effect on physical recovery and functional status, when compared to significant improvements over time, particularly during the first eight weeks post-hospital discharge. Both groups improved their physical endurance and HRQOL with a similar trajectory at 8 and 26 weeks.

Possible reasons for lack of difference between the groups were: 1) Natural recovery in this cohort overwhelmed any differences afforded by training; 2) Lack of compliance by intervention group participants, as exercise training was unsupervised except for three occasions during the eight weeks; 3) Prospective measurement of the control group at 8 and 26 weeks may have influenced outcomes by encouraging participation in exercise; 4) Training intensity was not adequate, although this was unlikely given that this was individualised, based on 6MWT results and progressed as able. Even at eight weeks the 6MWD was only approximately 408 metres suggesting that using 6MWD as a way of prescribing intensity was still adequate. Had the 6MWD been near to predicted normal (600 m) this might have suggested that other means of exercise training where intensity could be higher, for example, running and treadmill exercise, would have been required. Some of these issues are discussed in the 'study limitations' below.

This sample of survivors of a critical illness was broadly representative of the 83,000 patients admitted to public-sector general adult ICUs in Australia each year, in terms of APACHE II score (19.5 vs 15) and mechanical ventilation hours (91 vs 71), with our study inclusion criteria of ≥48 hours of ICU admission and ≥24 hours of mechanical ventilation probably accounting for these differences. (An additional 36,000 adults are admitted to ICUs in private hospitals but their clinical profile is different (APACHE II = 13; median mechanical ventilation hours = 44; ICU mortality = 2.7% vs 7.5%) [1]). The mortality rate for study participants at six months was 6% (11/183), within our *a priori *expectations of 10%, although we have no data on those participants who withdrew prior to (*n *= 12) or after the Week 1 assessment (*n *= 16) or were lost to follow-up (*n *= 7).

A recent systematic review [38] of 53 observational studies noted decreased HRQOL in survivors of a critical illness compared to age and gender-matched populations. In relation to other intervention studies from the United Kingdom, our participants at Week 1 and Week 26 were remarkably similar to a recent equivalent randomised clinical trial of nurse-led ICU follow-up clinics in three hospitals (median age = 57 years; 60% males; APACHE II = 19; ICU LOS was higher in our study, six vs. three days; mechanical ventilation hours not reported) [14]. Their intervention also included a manual-based, self-directed physical rehabilitation program from in-hospital to three months post-hospital discharge, and clinic appointments at three and nine months. The PCS at Week 1 was 32 in our study, compared to 33 (*n *= 286) in the U.K. study. At week 26, the PCS in our study was 43, compared to 40 (*n *= 220). Interestingly, this trial also did not demonstrate an intervention effect, and noted the possible reasons for this null finding as an ineffective intervention package, timing of the first intervention, no account for cognitive factors that may influence recovery, or too broad an inclusion criteria (particularly in relation to ICU LOS) [14].

In contrast, an earlier study of a six-week self-help rehabilitation manual with phone follow-up compared to usual care (ward visits, three post-discharge phone-calls, and ICU follow-up clinics at eight weeks and six months), demonstrated an effect on the PF at six months; scores for the control and intervention groups were 40 and 50 respectively (*n *= 44 and 58) [15]. These scores for the control group were again similar to our findings of approximately 42 for both groups. Only modest improvements on the PF were also noted from eight weeks to six months [15], again similar to our findings from 8 to 26 weeks. Neither of these two studies assessed walk function.

Overall, participants from both the control and intervention groups improved their 6MWT distance by 27% at 8 weeks and 39% at 26 weeks from the Week 1 assessments. These improvements of 89 and 120 metres compared favourably with increases from pulmonary rehabilitation programs for patients with moderate-severe lung disease (35 metres; 10% improvement) [17] and diffuse lung disease (34 metres) [39]. In an observational study of ARDS patients (*n *= 109), a median 6MWT distance of 396 metres at six months (*n *= 78; APACHE II = 23; ICU LOS = 25 days; mechanical ventilation = 21 days) compared favourably to the 430 metres in our sample of less sick patients [40].

The eight-week intervention of three home visits for at least one hour of supervised training, four telephone follow-ups, and an expected two to three unsupervised participant training sessions per week for the eight-week program was consistent with studies of COPD patients [41,42]. Recent clinical practice guidelines recommend high-intensity aerobic training (60 to 80% of peak effort) and strength training for COPD patients [43].

A small RCT of early physical therapy in ICU in combination with daily interruption of sedation demonstrated that the intervention group participants were 2.7 times more likely to return to independent functional status at hospital discharge. Median walking distance at hospital discharge for the intervention group (*n *= 49) was 33 metres (range 0 to 91 m), compared to 0 metres (0 to 30 m) for the control group (*n *= 55) [44]. Findings from a current single-site randomised study of a post-ICU and outpatient clinic rehabilitation program is anticipated to add further understanding to the effect of these types of interventions on function across the continuum of recovery [45].

A recent systematic review of 12 RCTs of cardiac rehabilitation noted superior adherence to a home-based program, with centre-based programs having sub-optimal participation because of access, dislike of groups and other commitments [16]. However, others have noted that an individually tailored exercise level was not sufficient to influence functional outcomes in hospitalised acute medical patients aged 65 years or older [46].

The burden for survivors of a critical illness has been well documented in many observational studies, where the recovery trajectory is often prolonged and sub-optimal [4]. Intervention studies with this clinical cohort are, however, less common. To our knowledge this was the first study internationally to use a home-based rehabilitation program with trainer visits in this patient group. An individualised, home-based program negates the need to attend an outpatient clinic located in a hospital on a regular basis. This is particularly important for individuals who reside in regional or rural areas but were treated in a metropolitan ICU, as well as those who choose not to or are unable to participate in hospital-based programs for other reasons such as lack of mobility. The provision of a program through local community health services would allow survivors of a critical illness to engage in the program regardless of place of residence and other mobility and access constraints.

### Study limitations

A number of limitations are noted. Although based on previous equivalent work [15], our hypothesised treatment 'effect' of a 10-point difference in the PF was optimistic, with no clinically or functionally important differences noted between groups at either post-intervention measurement; both groups improved by an average 12 points at eight weeks (not 5 and 15 points for the control and intervention groups, respectively). Effectiveness of the rehabilitation program may be improved by increasing the intensity, frequency and training support, but this requires further investigation, particularly in relation to participant safety in a home-based context. Importantly and similar to an earlier study, [14] our collective knowledge of physical rehabilitation in this cohort has advanced significantly since we designed our intervention, including a recent focus on in-ICU mobility [for example 47, 48, 49, 50].

Our target recruitment sample of 240 was not achieved despite screening almost 6,000 patients over 39 months, including a 12-month extension of the project from the grant funding body and inclusion of additional recruitment sites (recruitment ceased because of timeframe and funding constraints). Our screening data noted a significant proportion (28%) of patients admitted to the city-based recruitment ICUs resided outside metropolitan areas. Following analysis, and as noted above, the effect size and resulting sample size calculations were too small.

As detailed in Figure 1, large numbers of patients were excluded from this study. Approximately half of these exclusions were due to the patient not being suitable for rehabilitation (for example, palliative care), or having a condition that required different rehabilitation (for example, neurological dysfunction or cardiac disease where rehabilitation was provided). Some of the exclusions were the result of limitations of a research setting (for example, living too far away from a study hospital), and these individuals could benefit if an effective intervention is able to be identified.

The three assessment visits for the control group were in addition to 'usual care'. While this contact was unavoidable and may have had a placebo effect, any effect would reduce the apparent effectiveness of the intervention. The treatment effect was therefore measured relative to the control group in the study, while in practice the comparator would be usual care without assessment contact. Measurement of physical activity in a trial can also influence participant behaviour [51]. We have no way of knowing how participants in our control group responded to their 6MWT assessments, possibly changing their physical activity behaviour.

We were unable to objectively assess the compliance of training for the intervention group, and relied on self-reports of participants during trainer home visits and follow-up phone calls. Finally, given that we demonstrated no added effectiveness from the intervention compared to the control, the lack of an economic evaluation was of no practical consequence.

### Implications for practice

From a practice perspective, the benefits of a systematic approach and equitable access to post-ICU rehabilitation services remains unclear for facilitating the recovery of survivors to their optimal physical, psychological and social function. While not demonstrating effectiveness of the intervention, actual delivery of this home-based program was feasible for participants and trainers. If an effective intervention can be identified, then using a home-based approach may be of value for some individuals unable to attend hospital outpatient clinics because of location, travel limitations or other reasons.

### Recommendations for future research

Future research should explore strategies to increase the effect size of any proposed intervention, and implement appropriate screening processes to identify individuals who would benefit most from a rehabilitation program; that is those with demonstrated functional weakness or impairment.

## Conclusions

This study used a multi-centre randomised controlled trial design to examine the efficacy of a novel application of physical rehabilitation practices to an important but often heterogeneous group of patients-survivors of a critical illness. The study addressed outcomes that are meaningful for patients and society-functional ability and well-being following a critical illness, and also targeted a health problem that is likely to increase as the population ages, contributing to an area in which there are currently minimal rigorous intervention studies.

While these null findings noted no significant effect on physical recovery when compared to improvement over time, the results do provide a baseline to further develop and test interventions aimed at improving the recovery trajectories for survivors of a critical illness.

## Key messages

• Reviews of observational studies confirm significant physical and psychological sequelae for a substantial proportion of critical illness survivors.

• There a re mixed findings from the limited number of intervention studies that tested the effectiveness of physical rehabilitation programs for survivors of a critical illness, and no studies have tested a home-based program using trainer visits.

• This study demonstrated that a home-based physical rehabilitation program was no more effective than usual care in improving physical function or health-related quality of life for survivors of a critical illness at 6 months following hospital discharge.

• Further work is required to improve the effect of any interventions for survivors of a critical illness.

## Abbreviations

6MWT: Six Minute Walk Test; 8RM: 8-repetition maximum; APACHE: Acute Physiology and Chronic Health Evaluation; ARDS: Acute Respiratory Distress Syndrome; CONSORT: Consolidated Standards of Reporting Trials; COPD: Chronic Obstructive Pulmonary Disease; HRQOL: Health Related Quality of Life; LOS: Length of Stay; MCS: Mental Component Summary score (of SF-36); NBS: Norm-Based Scoring (of SF-36); PCS: Physical Component Summary score (of SF-36); PF: Physical Function scale (of SF-36); RCT: Randomised Controlled Trial; SAS: Statistical Analysis Software; SD: Standard Deviation; SF-36 (Medical Outcomes Study): Short Form-36 Health Survey.

## Competing interests

The authors declare that they have no competing interests.

## Authors' contributions

DE conceived the study and took responsibility for the design, implementation and reporting of the study. DE, SM, JA, LA and MK developed the study design and methods. All authors contributed to the conduct of the study including participant recruitment and follow-up, data collection and management. JA specifically led development of the exercise manual. MK, SM and DE contributed to the statistical analysis plan and power calculations. Analyses were conducted by PK and EB. DE, SM, JA, LM and MK had full access to all of the data in the study and take responsibility for the integrity of the data and the accuracy of the data analysis. PT was the project manager, and GL, PT, RF and EB acted as site coordinators. DE drafted the manuscript and all other authors critically revised it for important intellectual content and approved the final version for publication.

Author affiliations for PT and RF are for the time of the project.

All authors declare that they accept full responsibility for the conduct of the study and controlled the decision to publish. All authors declare that they have no conflicts of interest (financial or personal relationships or affiliations) that could influence our decisions, work, or the manuscript.
